# Supplementary figures and images for: Prevalence of Anti-SARS-CoV-2 Antibodies in Poznań, Poland, after the First Wave of the COVID-19 Pandemic
Source: Vaccines (Basel). 2021 May 21;9(6):541. doi: 10.3390/vaccines9060541 (PMC8223993; doi:10.3390/vaccines9060541)

Figure S1

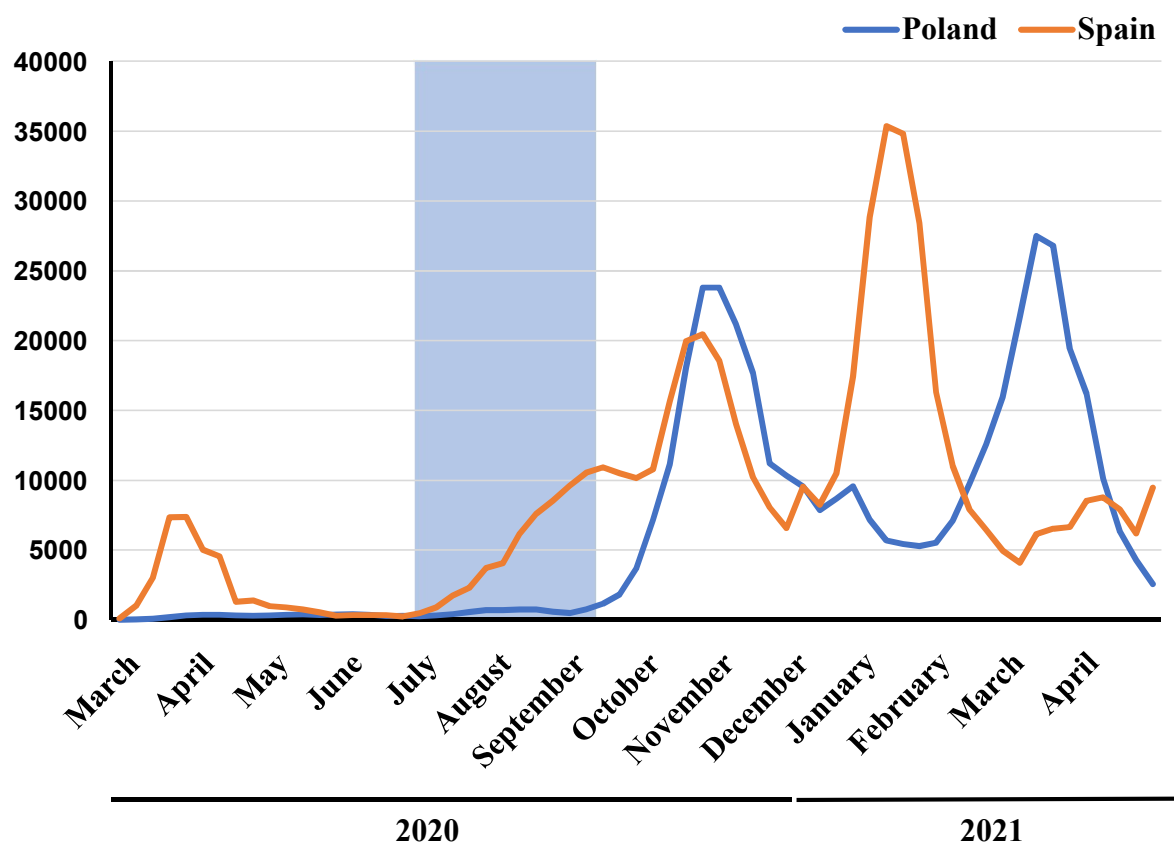

Supplement: Supplementary file 1 [file vaccines-09-00541-s001.zip › vaccines-1222872-SI.pdf]
